# Application of Large Language Models in Complex Clinical Cases: Cross-Sectional Evaluation Study

**DOI:** 10.2196/73941

**Published:** 2025-08-14

**Authors:** Yuanheng Huang, Guozhen Yang, Yahui Shen, Huiguo Chen, Weibin Wu, Xiaojun Li, Yonghui Wu, Kai Zhang, Jiannan Xu, Jian Zhang

**Affiliations:** 1 Department of Cardiothoracic Surgery, Third Affiliated Hospital of Sun Yat-sen University, 2693 Kaichuang Avenue, Huangpu District, Guangzhou, 510000, China, 86 13922192727, 86 82179042; 2 Department of Gynecologic Oncology, Shaanxi Provincial Cancer Hospital, Xi'an, China

**Keywords:** large language models, artificial intelligence, clinical decision support, complex medical cases, cross-sectional studies

## Abstract

**Background:**

Large language models (LLMs) have made significant advancements in natural language processing (NLP) and are gradually showing potential for application in the medical field. However, LLMs still face challenges in medicine.

**Objective:**

This study aims to evaluate the efficiency, accuracy, and cost of LLMs in handling complex medical cases and to assess their potential and applicability as tools for clinical decision support.

**Methods:**

We selected cases from the database of the Department of Cardiothoracic Surgery, the Third Affiliated Hospital of Sun Yat-sen University (2021‐2024), and conducted a multidimensional preliminary evaluation of the latest LLMs in clinical decision-making for complex cases. The evaluation included measuring the time taken for the LLMs to generate decision recommendations, Likert scores, and calculating decision costs to assess the execution efficiency, accuracy, and cost-effectiveness of the models.

**Results:**

A total of 80 complex cases were included in this study, and the performance of multiple LLMs in clinical decision-making was evaluated. Experts required 33.60 minutes on average (95% CI 32.57‐34.63), far longer than any LLM. GPTo1 (0.71, 95% CI 0.67‐0.74), GPT4o (0.88, 95% CI 0.83‐0.92), and Deepseek (0.94, 95% CI 0.90‐0.96) all finished under a minute without statistical differences. Although Kimi, Gemini, LLaMa3-8B, and LLaMa3-70B took 1.02‐3.20 minutes, they were still faster than experts. In terms of decision accuracy, Deepseek-R1 had the highest accuracy (mean Likert score=4.19), with no significant difference compared to GPTo1 (*P*=.699), and both performed significantly better than GPT4o, Kimi, Gemini, LLaMa3-70B, and LLaMa3-8B (*P*<.001). Deepseek-R1 and GPTo1 demonstrated the lowest hallucination rates—6/80 (8%) and 5/80 (6%), respectively—significantly outperforming GPT-4o (7/80, 9%), Kimi (10/80, 12%), and the Gemini and LLaMa3 models, which exhibited substantially higher rates ranging from 13/80 (16%) to 25/80 (31%). Regarding decision costs, all LLMs showed significantly lower costs than the Multidisciplinary Team**,** with open-source models such as Deepseek-R1 offering a zero direct cost advantage.

**Conclusions:**

GPTo1 and Deepseek-R1 show strong clinical potential, boosting efficiency, maintaining accuracy, and reducing costs. GPT4o and Kimi performed moderately, indicating suitability for broader clinical tasks. Further research is needed to validate LLaMa3 series and Gemini in clinical decision.

## Introduction

### Background

With the development of artificial intelligence (AI) and deep learning technologies, large language models (LLMs) have demonstrated remarkable potential across various fields, particularly in natural language processing (NLP) tasks such as summarization, paraphrasing, generating new text content, and writing program code [1,2]. Additionally, LLMs can act as personal assistants, helping users answer a wide range of questions.

The primary goal of LLMs was not initially to serve the medical field, but some studies have already shown that LLMs have significant potential in medicine [3,4]. For example, research has found that ChatGPT can pass the United States Medical Licensing Examination (USMLE) [5] and the Advanced Cardiovascular Life Support (ACLS) exam [6]. In addition to exam simulations, ChatGPT’s potential advantages in daily medical applications have also been confirmed, such as extracting information from electronic health records and assisting with literature searches [7]. However, the training knowledge base of LLMs may have issues such as incompleteness, information bias, or generation of misleading information, and the application of general-purpose LLMs in medicine has not yet undergone large-scale clinical studies. Therefore, it is essential to explore the application of LLMs in medicine in greater depth.

Previous studies have verified the diagnostic capabilities of GPT-3.5, GPT-4, and other LLMs [8-12], such as its ability to generate detailed differential diagnosis lists for common clinical cases. Additionally, research teams tested the GPT-4 ability to make accurate diagnoses from medical records [9], and the results showed that the generative AI GPT-4 chose the correct diagnosis as the primary diagnosis in nearly 40% of cases and provided the correct potential diagnosis in 64% of challenging cases. However, complex cases are common in hospitals, especially in cardiothoracic surgery. Due to population aging and insufficient medical resources, providing timely and high-quality clinical decision support has become a major challenge for health care systems worldwide. Delivering accurate and effective clinical decisions for complex cases typically requires substantial human, time, and money. Although some studies are currently exploring the application of LLMs in medicine, a recent review [13] published in a *Journal of the American Medical Association* subjournal of 519 studies assessing LLMs in the medical field found that only 5% of studies used real-world data. This exposes the limitations of current research and the potential disconnect from clinical reality. In this context, whether LLMs can provide effective clinical decision support for doctors in complex cases still requires further investigation.

### Objectives

Therefore, this study aimed to preliminarily evaluate the utility of LLMs in complex medical decision-making by assessing their efficiency, accuracy, and cost in real-world clinical cases, and exploring their practical potential in clinical decision support.

## Methods

### Study Design and Data Source

To ensure that the study is based on representative and unbiased real-world cases, we screened a total of 140 cases from the complex case database of the Department of Cardiothoracic Surgery of the Third Affiliated Hospital of Sun Yat-sen University (2021‐2024). These cases are sourced from the complex case database of a tertiary hospital, which is not publicly available, ensuring the accuracy of the study to the greatest extent and preventing any generic LLMs from being exposed to relevant data during their training, thus ensuring the objectivity and fairness of the evaluation. Most of the cases have a consistent structure, including medical history, current symptoms, examination methods and findings, diagnosis, and clinical decision. All cases were reviewed by an experienced physician, and only those that met the following criteria were included in the further analysis: the clinical case data records the entire diagnostic and treatment process, complex clinical case, and the cases are not duplicates. In this study, a complex case was defined as a patient scenario that disease involved at least 2 organ systems, required multidisciplinary input for diagnosis or treatment, and presented with conflicting or uncertain therapeutic pathways. These criteria were established by 2 senior cardiothoracic specialists.

### LLMs Evaluation

To evaluate the recently launched LLMs, we selected the following models for benchmarking: Deepseek-R1, GPTo1, GPT-4o, Kimi, Gemini, LLaMA3-70B, and LLaMA3-8B. Model setup and prompting strategy details are provided in Multimedia Appendix 1. The reviewers included 2 board-certified cardiothoracic surgeons, each with more than 10 years of clinical experience and active participation in weekly Multidisciplinary Team (MDT) discussions. Reviewers were blinded to the identity of the LLM models generating each response to minimize bias.

#### Clinical Decision Efficiency Evaluation

We evaluate the execution efficiency of Deepseek-R1, GPTo1, GPT4o, Kimi, Gemini, LLaMA3-70B, and LLaMA3-8B in clinical decision-making tasks. This is done by recording the time taken by each model from receiving the instructions to generating a complete decision recommendation and comparing it with the time taken by several experts to complete the same task.

#### Clinical Decision Accuracy Evaluation

Two independent clinical experts were invited to evaluate the clinical decision outputs generated by Deepseek-R1, GPTo1, GPT-4o, Kimi, Gemini, LLaMA3-70B, and LLaMA3-8B. The evaluations were based on the consistency with expert decisions, using a 5-point modified Likert scale. The scoring standards and the scale are presented in Table 1 [14,15].

**Table 1.** Scoring standards for the 5-point modified Likert scale.

Likert score	Relevance of decisions	Redundancy of decisions
1	Most of all relevant decisions were not mentioned.	All or most decisions were redundant or unjustified.
2	Some or many relevant decisions were not mentioned.	Some decisions were redundant or unjustified.
3	Most relevant decisions were mentioned.	Some decisions were redundant or unjustified.
4	Most relevant decisions were mentioned.	Few decisions were redundant or unjustified.
5	All relevant decisions were mentioned.	No redundant or unjustified decisions.

### Hallucination Evaluation

Each LLM output was reviewed by two independent clinical experts to identify statements that were factually incorrect, clinically implausible, or not supported by the case information. A hallucination was defined as a recommendation or rationale inconsistent with current clinical guidelines or contradicting the provided case data. The hallucination rate was calculated as the proportion of cases where at least one hallucinated item was present in the model’s response.

Each expert independently scored the output of each model, and the final score was the average of the two experts’ ratings. To ensure scoring consistency, we calculated the inter-rater reliability (Cohen kappa coefficient—a statistic that measures inter-rater agreement for categorical items while accounting for chance agreement) [16] to validate the reliability of the evaluation.

### Clinical Decision Cost Evaluation

The usage costs of each LLM were calculated and compared with the costs of the MDT. The MDT costs were calculated based on the hospital’s MDT fee.

### Statistical Analysis

Patient baseline characteristics were described using frequencies and percentages for categorical variables, and medians or means for continuous variables. For continuous variables with approximate normal distribution, Student *t* test (2-tailed) was used for comparisons; for nonnormally distributed continuous variables, the Mann-Whitney *U* test was used for comparisons between two groups, and the Kruskal-Wallis test was used for comparisons between three or more independent samples. For categorical data, Fisher exact test, chi-square test, or Wilcoxon signed-rank test was used. All statistical tests were 2-tailed, and a *P* value<.05 was considered statistically significant. Statistical analysis and plotting were performed using Python 3.13.1, with libraries including scipy.stats, pandas, seaborn, and matplotlib.

### Ethical Considerations

This study was approved by the ethics committee of the Third Affiliated Hospital of Sun Yat-sen University (approval ID II2025-257-03; approval date March 2025). Written informed consent was obtained from all participants. To protect privacy, personal identifiers were removed or coded before analysis, and all data were stored on an encrypted, password-protected server accessible only to the research team. Participants did not receive any financial or material compensation. All procedures involving human participants were conducted in accordance with the Declaration of Helsinki and relevant institutional guidelines.

## Results

### Baseline Characteristics

A total of 140 patients from the Cardiothoracic Surgery Department of the Third Affiliated Hospital of Sun Yat-sen University were initially included in the study. After excluding 60 patients who did not meet the inclusion criteria, 80 patients remained. The median age of the study population was 60 (IQR 14‐79) years, with 58 (72%) males and 22 (28%) females. The most common complex conditions were thoracic tumors combined with respiratory system disorders (25/80 cases, 31%), followed by thoracic tumors combined with circulatory system disorders (19/80 cases, 24%). As shown in Table 2, other conditions accounted for the remaining 45% (36/80 cases), and the median number of experts involved in the MDT was 7 (range 5‐9). Table S2 in Multimedia Appendix 1 illustrates 2 representative complex clinical cases, including the final expert consensus from MDT discussions and the corresponding responses generated by LLMs.

**Table 2.** Baseline characteristics of the study cohort.

Characteristics	Values (N=80)
Age (years), median (range)	60 (14‐79)
Sex, n (%)	
Male	58 (72)
Female	22 (28)
Complex conditions, n (%)	
Thoracic tumors+ respiratory disorders	25 (31)
Thoracic tumors+ circulatory disorders	19 (24)
Other conditions	36 (45)
Number of experts in MDT^a^, median (range)	7 (5-9)

a MDT: Multidisciplinary Team.

### Clinical Decision Efficiency Evaluation

**Figure 1.**
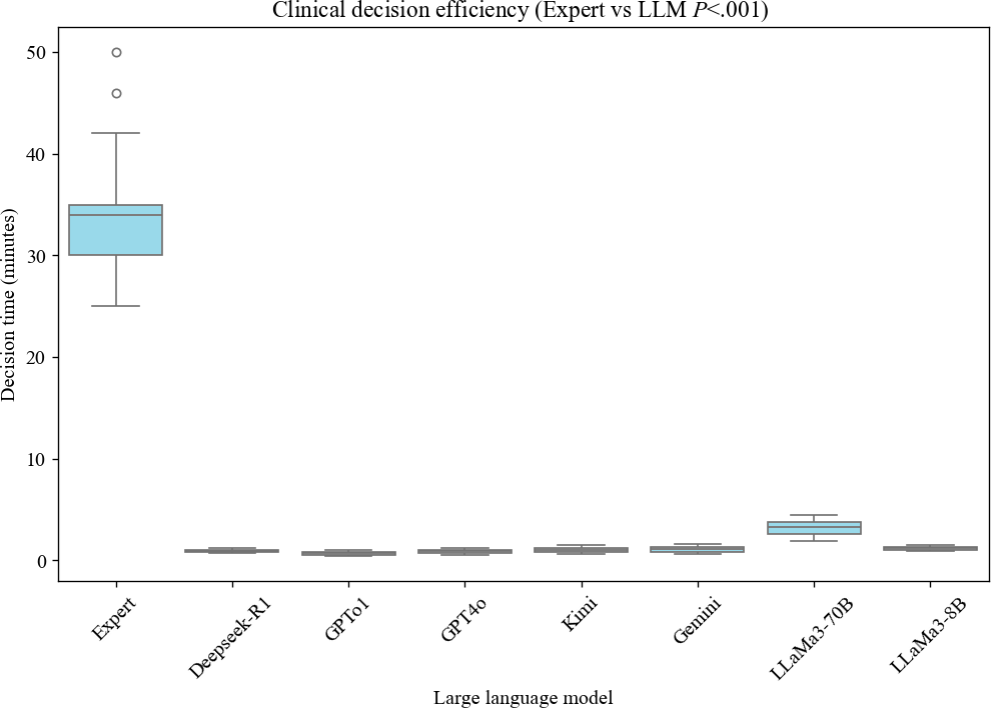
Clinical decision efficiency comparison between experts and large language models. LLM: large language model.

The data analysis revealed that the average decision-making time for the expert group was significantly longer than for all LLMs (*P*<.001), with a mean of 33.6 (95% CI 32.57‐34.63) minutes. In contrast, the decision-making time for LLMs was notably shorter. GPTo1 demonstrated the best decision-making efficiency, with an average time of only 0.71 (95% CI 0.67‐0.74) minutes, followed by GPT4o (0.88, 95% CI 0.83‐0.92 minutes) and Deepseek (0.94, 95% CI 0.90‐0.96 minutes), all of which completed decisions in under 1 minute. Kimi, Gemini, and LLaMa3-8B also exhibited relatively fast decision-making abilities (1.02‐1.18 minutes), although slightly slower than GPTo1 and GPT4o. LLaMa3-70B had the longest decision time at 3.20 (95% CI 3.04‐3.37) minutes, but it was still significantly better than the expert group’s 33.6 minutes. These results indicate that AI-driven LLMs significantly enhance the efficiency of clinical decision-making, as illustrated in Figure 1.

### Clinical Decision Accuracy Evaluation

**Figure 2.**
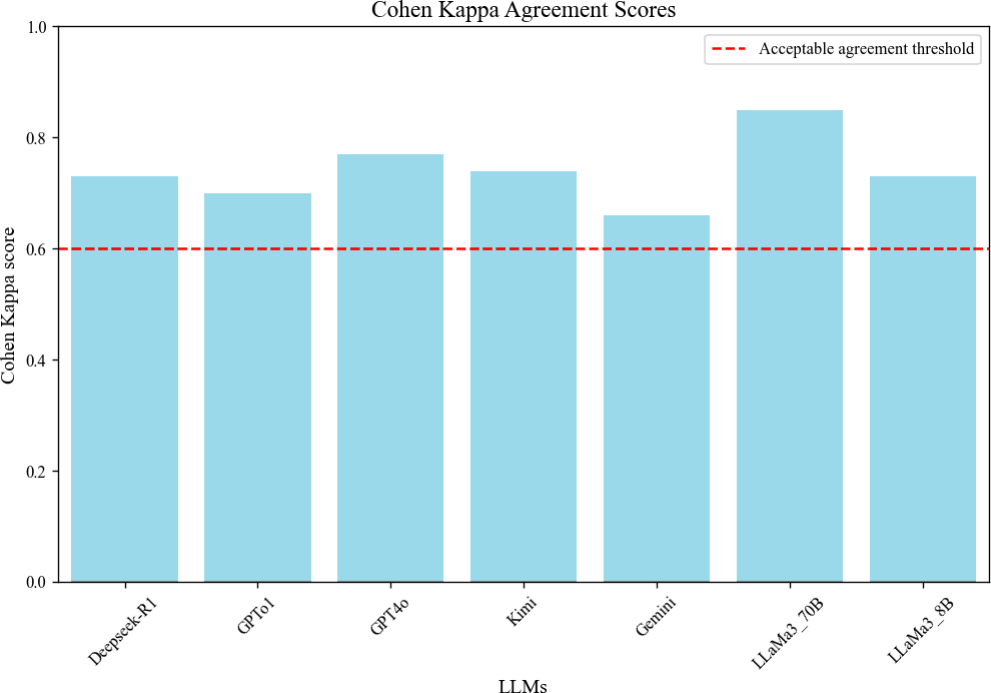
Cohen Kappa agreement between 2 senior clinical experts. LLMs: large language models.

According to the classification standards of Landis et al [16], which are based on Cohen kappa coefficient, the results indicate strong agreement between two senior independent clinical experts, with kappa values ranging from 0.66 to 0.85. This suggests a high level of consistency in model evaluation. Detailed results are presented in Figure 2.

**Figure 3.**
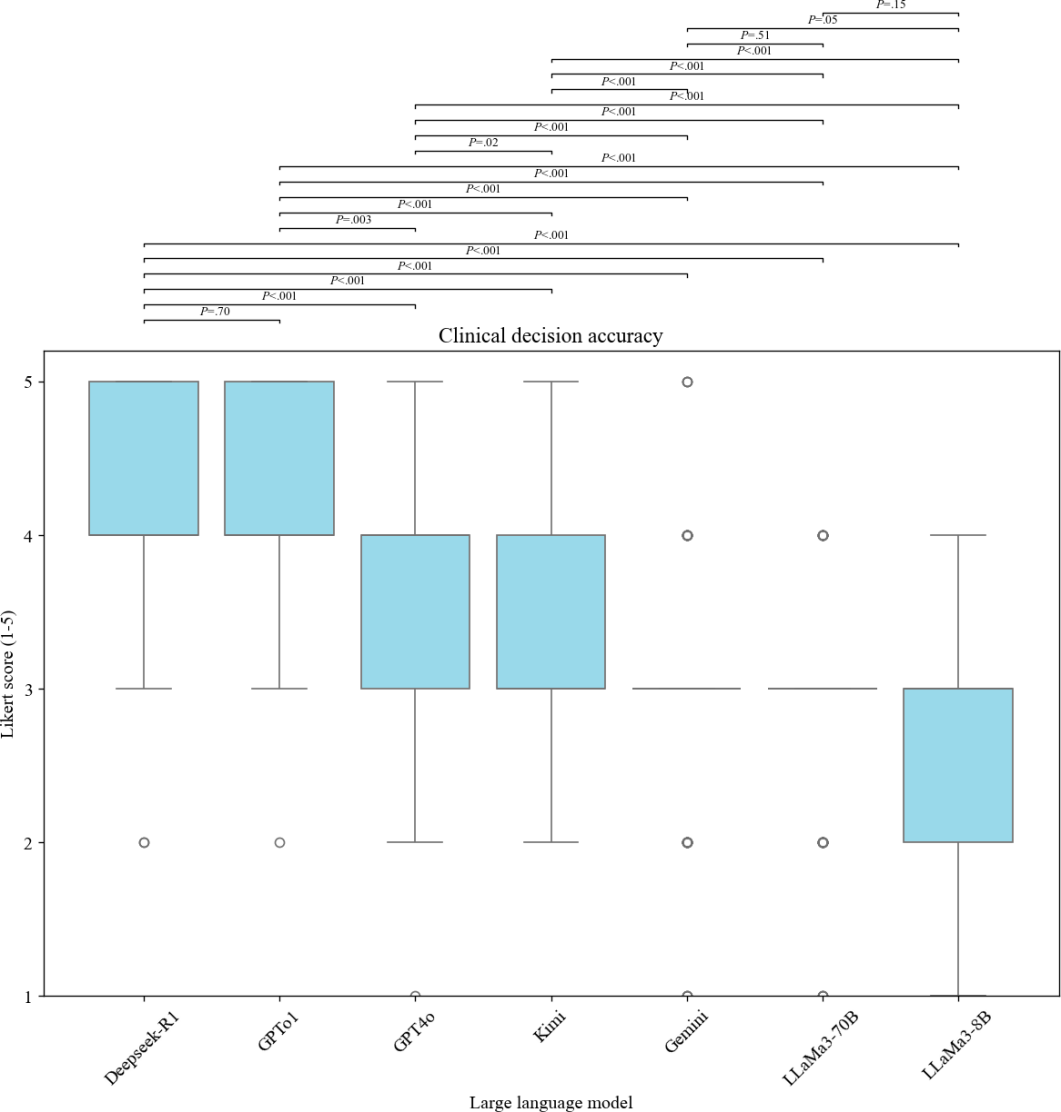
Clinical decision accuracy of large language models based on a 5-point Likert Scale.

As shown in Figure 3, pairwise comparisons in clinical decision accuracy assessment revealed that Deepseek-R1 achieved the highest accuracy, with a mean Likert score of 4.19 (95% CI 4.02‐4.35), significantly outperforming GPT4o, Kimi, Gemini, LLaMA3-70B, and LLaMA3-8B (all *P*<.001). However, no significant difference was found between Deepseek-R1 and GPTo1 (mean 4.15, 95% CI 3.99‐4.31; *P*=.70). GPTo1 performed comparably to Deepseek-R1 and significantly better than GPT4o (*P*=.003), Kimi, Gemini, LLaMa3-70B, and LLaMa3-8B (all *P*<.001). Both Deepseek-R1 and GPTo1 exhibited the highest Likert scores, approaching expert-level performance, indicating suitability for clinical decision tasks.

GPT-4o achieved moderate performance with a mean Likert score of 3.76 (95% CI 3.57‐3.95) but still significantly outperformed Kimi (*P*=.02), Gemini, LLaMa3-70B, and LLaMa3-8B (all *P*<.001), suggesting adequate feasibility for clinical decision-making. Kimi (mean 3.48, 95% CI 3.31‐3.64) had higher scores compared to Gemini, LLaMa3-70B, and LLaMa3-8B (all *P*<.001), although it demonstrated significantly lower decision-making ability than Deepseek-R1, GPTo1, and GPT4o.

Gemini (mean 2.96, 95% CI 2.78‐3.14) showed marginally better performance compared to LLaMa3-70B (mean 2.86, 95% CI 2.71‐3.02; *P*=.047), but no significant difference was observed between Gemini and LLaMa3-8B (mean 2.7, 95% CI 2.54‐2.86; *P*=.52). The LLaMa3 series consistently showed the lowest clinical decision accuracy, significantly below all other models (all *P*<.001).

**Figure 4.**
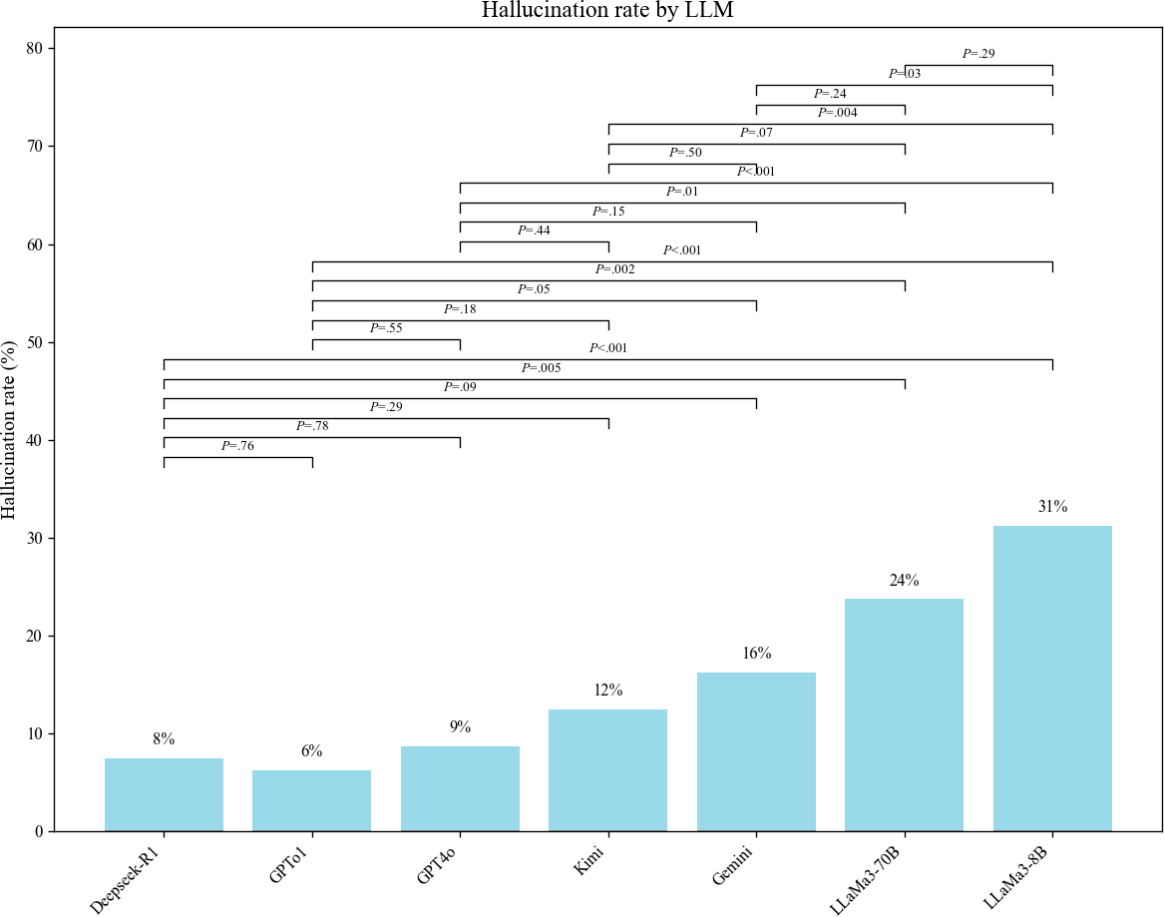
Comparison of hallucination rates among different large language models.

We further assessed the hallucination rates of LLMs and found that Deepseek-R1 and GPTo1 had the fewest hallucinations, with 6/80 (8%) and 5/80 (6%) cases, respectively. GPT-4o and Kimi followed, with 7/80 (9%) and 10/80 (12%) cases, respectively. In contrast, Gemini and both LLaMa3 variants demonstrated significantly higher hallucination rates, ranging from 13/80 (16%) to 25/80 (31%) (Figure 4.

These findings demonstrate that Deepseek-R1 and GPTo1 not only achieved the highest Likert scores but also exhibited the lowest hallucination rates, underscoring their strong potential as AI-assisted tools for clinical decision-making. GPT-4o exhibited moderate yet consistent performance, supporting its applicability to more complex clinical scenarios. Kimi demonstrated intermediate performance, potentially useful in selected clinical situations but requiring further optimization. Conversely, Gemini, LLaMa3-70B, and LLaMa3-8B showed relatively poor performance, limiting their immediate clinical utility and highlighting the need for substantial improvement.

### Clinical Decision Cost Evaluation

**Figure 5.**
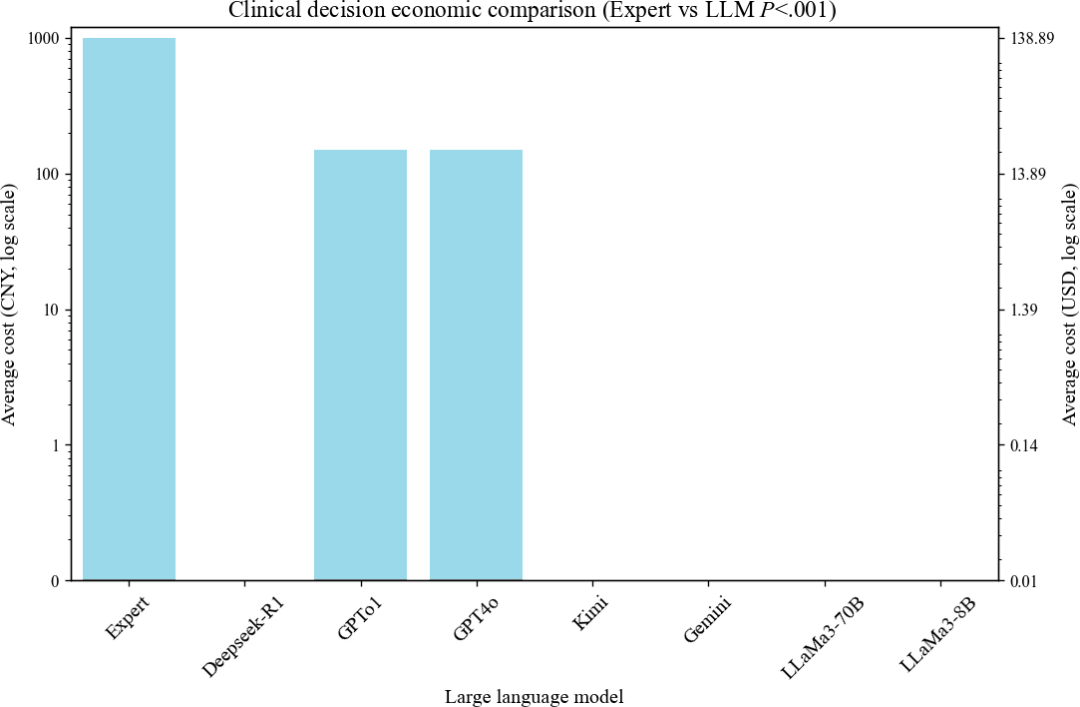
Cost comparison of clinical decision-making: experts versus large language models. CNY: Chinese Yuan Renminbi; LLM: large language model.

In this study, we compared clinical decision-making costs between MDT discussions and various LLMs (Figure 5). Our analysis revealed that the mean cost per MDT discussion conducted by experts was approximately 1000 Chinese Yuan Renminbi (about US $140), significantly higher than the decision-making costs associated with all evaluated LLMs (*P*<.001).

Within the LLM group, GPT4o and GPTo1 incurred direct costs of approximately 150 Chinese Yuan Renminbi (about US $20 for the Plus version), representing an 85% cost reduction compared to expert-led MDT discussions, thus demonstrating significant economic advantages. Additionally, Kimi and Gemini are proprietary models currently available without charge, whereas Deepseek-R1, LLaMa3-8B, and LLaMa3-70B are open-source models suitable for clinical decision support. These open-source LLMs potentially incur zero direct costs.

## Discussion

### Principal Findings

This study provides a preliminary assessment demonstrating that LLMs can significantly enhance the efficiency of clinical decision-making in complex clinical cases, while maintaining high decision accuracy and substantially reducing costs compared to traditional MDT discussions.

### Performance and Potential Clinical Utility of LLMs

Our study results demonstrate that, compared to human experts, all LLMs significantly shortened decision-making time. The expert group had an average decision time of 33.6 (95% CI 32.57‐34.63) minutes per case and involved a median of 7 clinical experts in the MDT discussion, while the most efficient model, GPTo1, took only 0.71 minutes, followed by GPT4o (0.88 minutes) and Deepseek (0.94 minutes). Even the longest decision time, observed in LLaMa3-70B, was only 3.20 minutes (which may be related to the insufficient GPU memory of the workstation in this study), still much faster than the expert group. Previous studies [17] also suggest that AI, through automating parts of the decision process, maintains accuracy in 88% of cases and provides quick, real-time feedback, thus improving efficiency and saving significant time and resources. This result highlights the potential application value of AI-assisted decision tools in high-demand clinical environments, especially in scenarios requiring rapid decision-making. Furthermore, the time differences among LLMs indicate that optimizing model architecture and inference time is critical to enhancing the real-time application capabilities of clinical decision-making.

In addition to improving decision efficiency, the reliability and accuracy of LLMs in clinical applications are equally crucial. This study found that Deepseek-R1 and GPTo1 performed the best in clinical decision accuracy, with decision levels comparable to that of human experts. However, there were still significant performance differences among the models. GPT-4o and Kimi showed moderate performance, suggesting that they may be suitable for general clinical tasks. For example, the accuracy of Gemini, LLaMa3-70B, and LLaMa3-8B was significantly lower, indicating limited applicability in critical clinical decision-making. Notably, Deepseek-R1 and GPTo1 demonstrated the lowest hallucination rates (8% and 6%, respectively), showing significantly better performance compared to GPT-4o (9%), Kimi (12%), and the Gemini and LLaMa3 models, whose rates ranged from 16% to 31%. These results underscore significant variability in content reliability among LLMs, aligning with prior studies [18] that reported hallucination rates ranging from 29% to 91% across different models. Interestingly, reasoning-optimized models such as Deepseek-R1 and GPTo1 showed a marked reduction in hallucinations, suggesting they may be more suitable for high-stakes medical applications where factual accuracy is critical. Beyond quantitative metrics, our study found qualitative differences in the reasoning styles of different LLMs. For example, Deepseek-R1 and GPTo1 tended to follow structured, guideline-concordant approaches, often closely mirroring the logic used by human MDT experts. GPT-4o and Kimi occasionally generated broader differential diagnoses. In contrast, Gemini, LLaMa3-70B, and LLaMa3-8B responses included more redundant or loosely justified recommendations. These observations are preliminary and illustrative. A more comprehensive and systematic comparison of LLM reasoning will be conducted in future studies. These findings reaffirm that LLMs require rigorous validation before being applied to clinical decision-making. Our findings are consistent with recent studies by other researchers [11], which suggest that LLMs can serve as auxiliary tools to provide reference advice to clinical medical experts, rather than completely replacing human experts.

Currently, MDT discussions are still in their early stages in China [19]. High costs remain one of the barriers to their widespread application. Studies show that the average cost of each MDT discussion by experts is approximately 1000 Chinese Yuan Renminbi (about US $140) in a tier 3 grade A hospital in China, while the decision cost for GPT4o and GPTo1 is only around 150 Chinese yuan renminbi (Plus subscription version US $19.99), representing an 85% cost reduction. Moreover, Kimi and Gemini, as closed-source models, are currently available for free, while Deepseek-R1, LLaMa3-8B, and LLaMa3-70B are open-source models with the potential advantage of zero direct costs. This makes open-source LLMs more promising in environments with limited health care resources. Although LLMs show significant advantages in terms of economic efficiency, their clinical application still needs to consider indirect costs, such as equipment costs, model fine-tuning, and ethical oversight.

### Risks and Deployment Strategies

It is also important to address privacy and security concerns when deploying LLMs in health care settings. In this study, some open-source models were deployed locally in a closed-loop environment without internet access or external application programming interface calls, effectively minimizing the risk of patient data leakage. In contrast, proprietary models such as GPT-4o, Kimi, and Gemini required data to be submitted via HTTPS after anonymization, which, despite encryption, still introduces potential risks of data exposure during transmission. For future clinical deployment, it is essential to implement privacy-preserving strategies such as on-premise inference, secure application programming interface gateways, and strict data de-identification.

While LLMs can significantly improve decision efficiency, maintain high diagnostic accuracy, and drastically reduce costs, some experts remain concerned about the potential misuse of LLMs and the lack of supervision [20]. At present, LLMs still have some imperfections, such as biases and hallucinations [21], which may be related to the Transformer architecture itself [22,23]. To address these challenges, several mitigation strategies should be considered, including deploying LLMs under expert supervision, conducting fine-tuning based on medical-specific datasets, and incorporating human-in-the-loop mechanisms to ensure clinical relevance and safety. Establishing robust ethical and governance frameworks is also imperative to support responsible and transparent deployment. In the short term, continued involvement of human experts remains critical, and the most pragmatic approach is a collaborative model in which LLMs assist clinicians rather than operate independently or autonomously.

### Limitations

This study also has some limitations. First, our evaluation focused on LLMs’ execution efficiency, decision accuracy, and costs, without in-depth exploration of the models’ limitations, interpretability, and ethical implications. Future studies will adopt more comprehensive evaluation frameworks, such as Transparent Reporting of a multivariable prediction model for Individual Prognosis or Diagnosis-LLM extension [24], to incorporate additional dimensions including reproducibility, bias, and potential harm. Second, the data used in this study were obtained from a single institution, necessitating further external validation to assess the generalizability of LLMs across diverse health care settings. Third, we did not explore the integration of LLMs into hospital information systems in this study. While LLMs demonstrate significant transformative potential in clinical decision-making, further clinical trials and real-world validation are needed before their formal adoption in practice.

### Conclusions

The study indicates that LLMs, particularly GPTo1 and Deepseek-R1, have immense potential in clinical decision-making, significantly improving efficiency, maintaining high diagnostic accuracy, and reducing costs. These models can serve as powerful auxiliary decision-making tools. GPT-4o and Kimi demonstrated moderate performance, suggesting that they may be suitable for general clinical tasks. However, the application of the LLaMa3 series and Gemini in clinical decision-making requires further investigation.

## Supplementary material

10.2196/73941 Multimedia Appendix 1 Evaluation setup and output comparison of various large language models in complex clinical cases.

## Abbreviations

ACLS: Advanced Cardiovascular Life Support
AI: artificial intelligence
LLM: large language model
MDT: Multidisciplinary Team
USMLE: United States Medical Licensing Examination
