# Supplementary material for: Application of Large Language Models in Complex Clinical Cases: Cross-Sectional Evaluation Study
Source: JMIR Med Inform. 2025 Aug 14;13:e73941. doi: 10.2196/73941 (PMC12501899; doi:10.2196/73941)
Supplement: Multimedia Appendix 1 [file medinform-v13-e73941-s001.docx]

**Model configuration and Prompting Strategy**

The same prompt was used for all models:

"Imagine you are an experienced clinical expert with solid knowledge in the medical field, and you are participating in an MDT (Multidisciplinary Team) consultation. How would you make the best clinical decision for this case? Please try to explain in detail."

The open-source model parameters are as follows: temperature = 0.6, top_P= 0.9, and maximum sequence length = 2048. These hyperparameters were selected based on LM Studio's recommended configuration and prior research experience [1] to ensure optimal performance during inference.

These open-source models were deployed as base models (i.e., non-instruction-tuned) using the Q4_K_M quantized format, which enables efficient local inference while maintaining acceptable performance. Open-source model environment, Platform: Windows 11 (Workstation version), Environment: LM studio v0.3.10, CPU: Intel i9 13900K, RAM: 128GB, GPU (Graphics Processing Unit): NVIDIA RTX 4090.

**Table S1.** A list of the recent LLMs.

| **Model** | **Description** |
| --- | --- |
| Deepseek-R1[2] | Latest model from Deepseek. |
| GPTo1[3] & 4o[4] | Latest iterations of OpenAI's GPT series. |
| Kimi[5] | Recent models from Moonshot AI. |
| Gemini[6] | Google's recent large language model. |
| LLaMa3-70B[7] and LLaMa3-8B[8] | Different sizes of Meta's open-source model. |

**Table S2.** Expert Recommendations vs LLM-Generated Management Recommendations.

| **Patient Case** | **Summary of Clinical Case** | **Expert MDT Recommendation** | **LLM-Generated Recommendations** |
| --- | --- | --- | --- |
| Left thoracic soft tissue sarcoma, Type II respiratory failure | 2-year dyspnea history, left lung mass invading mediastinum, severe obstructive-mixed ventilation defect, biopsy: mesenchymal spindle cell tumor | Not a surgical candidate due to poor lung function; recommend further IHC + NGS to subtype sarcoma, then initiate oncology treatment | **Deepseek-R1:** Complete IHC/FISH (e.g., SYT-SSX fusion transcripts), correct type II RF, consider palliative chemo/radiation. |
|  |  |  | **GPTo1:** Wait for IHC, assess resectability, individualize based on lung reserve. |
|  |  |  | **GPT4o:** IHC confirmation, imaging for staging, optimize respiratory support. |
|  |  |  | **Kimi:** Oxygen, NIV if needed; defer surgery; consider chemo/radiation after pathology. |
|  |  |  | **Gemini:** IHC for classification, consider surgery if resectable. |
|  |  |  | **LLaMa3-70B:** IHC subtyping, supportive care, assess for chemo/surgery. |
|  |  |  | **LLaMa3-8B:** Suggest IHC; decide treatment based on pathology + symptoms. |
| Suspected early-stage lung cancer + Eisenmenger syndrome | A nodule in the right lower lobe of the lung was detected more than 8 months ago. PET-CT: suspected malignancy, no lymph node spread; congenital heart failure, severe PAH (PASP > 120), severe mixed ventilatory defect, hypoxemia on oxygen, arrhythmias | Surgery not advised due to high anesthesia risk and limited survival gain; recommend completing biopsy and considering radiotherapy | **Deepseek-R1:** Optimize PAH + heart function, treat COPD/infection, consider SBRT if cancer confirmed. |
|  |  |  | **GPTo1:** Optimize heart/lung status, assess SBRT/targeted/IO if cancer confirmed. |
|  |  |  | **GPT4o:** Prioritize QoL and non-surgical options; consider targeted or IO therapy. |
|  |  |  | **Kimi:** Emphasize pathology + function evaluation before surgery decision. |
|  |  |  | **Gemini:** Biopsy or surgical resection to confirm diagnosis; MDT to plan treatment. |
|  |  |  | **LLaMa3-8B:** Cardiac imaging, biopsy, consider non-invasive options. |
|  |  |  | **LLaMa3-70B:** Antihypertensives, biopsy, drug/supportive treatment suggested. |
| Aberration: IHC: Immunohistochemistry IO: Immunotherapy NGS: Next-Generation Sequencing NIV: Non-Invasive Ventilation PAH: Pulmonary Arterial Hypertension QoL: Quality of Life RF: Respiratory Failure SBRT: Stereotactic Body Radiotherapy | | | |
|  |  |  |  |
|  |  |  |  |
|  |  |  |  |
|  |  |  |  |

**References:**

1. Sandmann, S., et al. Systematic analysis of ChatGPT, Google search and Llama 2 for clinical decision support tasks. Nat Commun. 2024. 15(1): p. 2050. doi: 10.1038/s41467-024-46411-8. PMID: 38448475

2. Deepseek. *Deepseek Chat*. 2025; Available from: <https://chat.deepseek.com/>.

3. OpenAI. *ChatGPT - GPTo1*. 2025; Available from: <https://chatgpt.com/>.

4. OpenAI. *ChatGPT - GPT-4o*. 2025; Available from: <https://chatgpt.com/>.

5. AI, M. *Kimi Chat*. 2025; Available from: <https://kimi.moonshot.cn/>.

6. Google. *Gemini*. 2025; Available from: <https://gemini.google.com/app>.

7. MetaAI. *LLaMA 3 - 70B*. 2025; Available from: <https://www.llama.com/>.

8. MetaAI. *LLaMA 3 - 8B*. 2025; Available from: <https://www.llama.com/>.
